# Neutralizing activity against Omicron subvariants BA.1, BA.2, and BA.4/5 following the third SARS-CoV-2 vaccination in cancer patients undergoing chemotherapy

**DOI:** 10.1016/j.clinsp.2025.100757

**Published:** 2025-08-20

**Authors:** Jina Yun, Bora Kim, Hyuk Kim, Sung Hee Lim, Seong Hyeok Choi, Ji Youn Kim, Hyun Jung Kim, Seong Kyu Park

**Affiliations:** a Division of Hematology-Oncology, Department of Medicine, Soonchunhyang University, Bucheon Hospital, Bucheon, Gyeonggi-do, South Korea; b Division of Hematology/Oncology, Department of Medicine, Samsung Medical Center, Sungkyunkwan University School of Medicine, Gangnam-gu, Seoul, South Korea

**Keywords:** Cancer, Omicron subvariants, Neutralizing antibody, SARS-CoV-2, COVID-19 Vaccine

## Abstract

**Objectives:**

Despite the availability of bivalent vaccines targeting both the ancestral SARS-CoV-2 strain and Omicron subvariants, vaccination rates remain low in South Korea. This study aims to evaluate the neutralizing activity against Omicron subvariants following the third SARS-CoV-2 vaccination in patients undergoing chemotherapy, as well as to assess the need for additional vaccinations.

**Methods:**

Between April and November 2022, the authors assessed the neutralizing activity of the third SARS-CoV-2 vaccine dose in 63 patients undergoing chemotherapy using an ELISA-based surrogate Virus Neutralization Test (sVNT). The authors examined the influence of factors such as prior COVID-19 infection, cancer type (solid vs. hematologic malignancy), type of chemotherapy regimen (cytotoxic chemotherapy, targeted therapy, immune checkpoint inhibitors), and vaccine type (homologous vector, homologous mRNA, heterologous) on neutralizing activity.

**Results:**

Among the 57 patients included in the analysis, 26 (45.6 %) had a history of SARS-CoV-2 infection. Patients with hematologic cancers exhibited lower neutralizing antibody responses compared to those with solid tumors for Omicron subvariants BA.1 (24.44 % vs. 71.68 %, *p* = 0.020), BA.2 (48.22 % vs. 94.59 %, *p* = 0.006), and BA.4/5 (24.76 % vs. 78.06 %, *p* = 0.046). Among uninfected patients (*n* = 31), sVNT inhibition scores were significantly lower across all subvariants (e.g., 5.89 % for BA.1 vs. 58.11 % in infected, *p* = 0.0025). Treatment delays due to infection were observed in 9 patients (17.75 days on average). Although no deaths were directly attributed to infection, one patient died due to disease progression following a treatment delay caused by the infection.

**Conclusions:**

Patients undergoing chemotherapy displayed weak neutralizing responses against Omicron subvariants, especially those with hematologic cancers or no prior infection. Consequently, it is crucial to actively recommend additional vaccinations with bivalent or newly developed vaccines for these vulnerable patients.

## Highlights

• COVID-19 bivalent vaccine uptake in Korea is low at 12.95 %.
• Cancer patients with no history of Omicron infection exhibit low antibody titers.
• Hematologic cancer patients exhibit the lowest immune response.
• These high-risk patients need additional vaccinations.
• Stronger recommendations are needed for these individuals.

## Introduction

Since its initial identification on January 30, 2022, the Omicron variant of SARS-CoV-2, initially classified as BA.1, has evolved into subvariants such as BA.2 and BA.4/5, becoming the predominant strain globally. Although the Omicron variant has not significantly increased mortality rates, the continuous emergence of new subvariants has sustained public health concerns. In response, bivalent vaccines targeting both the ancestral SARS-CoV-2 strain and specific Omicron subvariants (BA.4 and BA.5) were developed in 2022–2023, led by Pfizer-BioNTech and Moderna. As of August 28, 2023, the basic vaccination rate against SARS-CoV-2 in South Korea remained robust at 87 %; however, the uptake of the bivalent vaccine, introduced in October 2022, was only 12.95 %.^1^ This disparity in vaccination rates reflects societal and psychological resistance, likely driven by concerns over vaccine efficacy and safety. Additionally, the rapid evolution of the virus compared to the slower pace of vaccine development has further fueled apprehensions. Recent studies, including Poh et al. (2022), have demonstrated that heterologous mRNA booster regimens, such as BNT162b2 followed by mRNA-1273, elicit significantly higher neutralizing antibody responses against Omicron variants compared to homologous regimens, particularly in older adults, suggesting potential benefits for vulnerable populations like cancer patients undergoing chemotherapy.^2^ For immunocompromised groups, such as cancer patients, infections caused by these evolving variants can disrupt ongoing chemotherapy and potentially exacerbate disease progression, making it crucial to reduce infection rates in this population.

This study aims to assess the effectiveness of the third SARS-CoV-2 vaccine dose in neutralizing Omicron subvariants BA.1, BA.2, and BA.4/5 in cancer patients undergoing chemotherapy and to determine the necessity of additional booster doses to protect this vulnerable group.

## Materials and methods

### Study design and participants

**Fig. 1:**
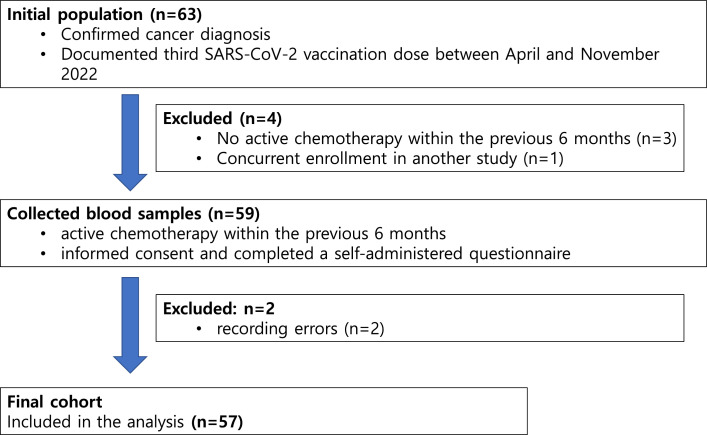
The flow chart of cohort selection.

This cross-sectional study collected blood samples from 59 cancer patients undergoing active chemotherapy, all of whom had received a third SARS-CoV-2 vaccination between April and November 2022 (Fig. 1). Blood samples were collected at least four months after the third dose. The analysis was conducted using a commercial surrogate Virus Neutralization Test (sVNT) kit (Genscript Biotech Corporation, Piscataway, NJ, USA). All participants provided informed consent and completed a self-administered questionnaire that gathered information on sex, age, vaccination date, vaccine type, history of COVID-19 infection, confirmed date of infection, and current chemotherapy status. Additionally, the authors obtained demographic data, oncological characteristics, and detailed information about chemotherapy (including treatment schedules, blood tests, and radiological results) from medical records. The authors compared sVNT values for Omicron subvariants (BA.1, BA.2, and BA.4/5) between patients who had contracted COVID-19 and those who had not, and further stratified these comparisons by tumor type, distinguishing between patients with solid tumors and those with hematologic malignancies.

### Serological assays

Neutralizing activity against Omicron subvariants (BA.1, BA.2, and BA.4/5) was assessed using ELISA-based sVNT. These subvariants displayed distinct amino acid changes in their spike receptor binding domain proteins. Specifically, BA.1 had the following mutations: G339D, S371L, S373P, S375F, K417N, N440K, G446S, S477N, T478K, E484A, Q493R, G496S, Q498R, N501Y, Y505H, and T547K. BA.2 featured mutations including G339D, S371F, S373P, S376A, D405N, R408S, K417N, N440K, S477N, T478K, E484A, Q493R, Q498R, N501Y, and Y505H. BA.4/5 had mutations such as G339D, S371F, S373P, S375F, T376A, D405N, R408S, K417N, N440K, L452R, S477N, T478K, E484A, F486V, Q498R, N501Y, and Y505H.The sVNT assay measures the inhibition of spike protein-receptor binding, with results expressed as percentage inhibition. The percentage inhibition was calculated as [1 − (OD of sample / OD of negative control)] × 100, where OD is optical density. A threshold of ≥68 % inhibition was used to indicate sufficient protection against COVID-19, as validated in prior studies.^3^^,^^4^

### Statistical analysis

Statistical analyses were conducted using GraphPad Prism software (version 9.3.1, San Diego, CA) and SPSS (version 27.0, Chicago, IL). For comparisons of continuous variables between two independent groups, either independent two-sample *t*-tests (for normally distributed data) or Wilcoxon rank sum tests (Mann-Whitney *U* test, for non-normally distributed data) were utilized. For comparisons involving continuous variables across three or more independent groups, one-way ANOVA (for normally distributed data) or the nonparametric Kruskal-Wallis test (for non-normally distributed data) was employed. Chi-Square tests were used for categorical variables. Paired *t*-tests were utilized for within-subject comparisons. All tests were two-tailed, and a p-value of < 0.05 was considered statistically significant.

### Ethics statement

The study was approved by the Institutional Review Board (IRB) of Soonchunhyang University Bucheon Hospital (IRB n° 2022–11–028), South Korea. Written informed consent was obtained from all participants.

This study adhered to the Strengthening the Reporting of Observational Studies in Epidemiology (STROBE) guidelines.

## Results

### Baseline characteristics

Of the 63 cancer patients initially included in this study, six were excluded due to recording errors, lack of chemotherapy, or concurrent enrollment in another study, resulting in a final cohort of 57 patients (Fig. 1). The mean age of the participants was 62.02 ± 10.98 years (range: 18‒79), with 59.7 % being male. All participants had received a third dose of the SARS-CoV-2 vaccine between January 21, 2021, and August 8, 2022. Among them, 49.1 % received a heterologous vaccine, 47.4 % received a homologous mRNA vaccine, and 3.5 % received a homologous vector vaccine (Table 1). The study population's clinical characteristics, including age, sex, infection status, cancer type, and chemotherapy regimens, were further stratified by vaccine type (homologous vector, homologous mRNA, and heterologous vaccines) to explore potential differences across vaccine regimens (Supplementary Table 1). Notably, patients receiving homologous vector vaccines (*n* = 2) were all diagnosed with hematologic malignancies, and a significant age difference was observed across vaccine types (*p* = 0.0166), though the small sample size limits further interpretation. The cohort included 64.2 % with solid malignancies (*n* = 36) and 36.8 % with hematologic malignancies (*n* = 21), all of whom were undergoing active chemotherapy within six months prior to sampling. The types of chemotherapy included 29.9 % cytotoxic therapy, 22.8 % targeted therapy, 5.3 % Immune Checkpoint Inhibitors (ICI), 31.6 % combined targeted therapy and chemotherapy, and 5.3 % combined ICI and chemotherapy.

**Table 1:** Clinical characteristics of the study population at baseline.

Characteristic	*n* = 57
**Age, years** (Mean ± SD (range))	62.02 ± 10.98 (18‒79)
**Sex**	
Male	34 (59.65 %)
Female	23 (40.35 %)
**SARS-Cov-2 Vaccine**	
Homologous vector vaccine	2 (3.51 %)
Homologous mRNA vaccine	27 (47.37 %)
Heterologous vaccine	28 (49.12 %)
**Solid malignancies**	36 (63.16 %)
Gastrointestinal cancer	17 (29.82 %)
Lung cancer	9 (15.79 %)
Gynecological cancer	4 (7.02 %)
Genitourinary cancer	4 (7.02 %)
Sarcoma	1 (1.75 %)
Hepatocellular carcinoma	1 (1.75 %)
**Hematologic malignancies**	21 (36.84 %)
Multiple myeloma	4 (7.02 %)
Lymphoma	7 (12.28 %)
Chronic lymphocytic leukemia	1 (1.75 %)
Chronic myeloid leukemia	4 (7.02 %)
Myelodysplastic syndrome or myeloproliferative neoplasms	5 (8.33 %)
**Ongoing chemotherapy**	57 (100.00 %)
Cytotoxic chemotherapy	17 (29.82 %)
Targeted therapy	13 (22.81 %)
Immune checkpoint inhibitor	3 (5.26 %)
Targeted therapy + chemotherapy	18 (31.58 %)
Immune checkpoint inhibitor + chemotherapy	3 (5.26 %)
Others	3 (5.26 %)

### Vaccine effectiveness

Among the 57 patients, 26 (45.6 %) tested positive for SARS-CoV-2 between January 31, 2022, and October 19, 2022, as confirmed by PCR tests conducted at public health centers or Soonchunhyang University Bucheon Hospital. This period coincided with near-total detection of the Omicron variant both domestically and internationally.^5^ The average interval between the third vaccination and confirmed infection was 109.96 ± 62.9 days. There were no significant differences between the infected and uninfected groups with respect to age, gender, vaccine type, cancer type, or type of chemotherapy (Table 2).

**Table 2:** Clinical characteristics of the study population by group.

Characteristic	Uninfected (*n* = 31)	Infected (*n* = 26)	p-value
**Sex (Male%)**	20 (64.52 %)	14 (53.85 %)	0.4113^b^
**SARS-Cov-2 Vaccine**			0.4651^b^
Homologous vector vaccine	1 (2.94 %)	1 (3.85 %)	
Homologous mRNA vaccine	17 (54.84 %)	10 (38.46 %)	
Heterologous vaccine	13 (41.94 %)	15 (57.69 %)	
**Solid malignancies**	22 (70.97 %)	14 (53.85 %)	0.2896^b^
**Hematologic malignancies**	9 (29.03 %)	12 (46.15 %)	
**Vaccination-sample interval (Q1, Q3)**	230 (174.5, 266.5)	203.5 (159.75, 236.25)	0.2866^a^
**Chemotherapeutic agents**			0.4275^b^
Cytotoxic chemotherapy	9 (29.03 %)	8 (30.77 %)	
Targeted therapy	5 (16.13 %)	8 (30.77 %)	
Immune checkpoint inhibitor (ICI)	1 (3.23 %)	2 (7.69 %)	
Targeted therapy + chemotherapy	11 (35.48 %)	7 (26.92 %)	
ICI + chemotherapy	3 (9.68 %)	0 (0 %)	
Others	2 (6.45 %)	1 (3.85 %)	

a p-value = Two sample *t*-test for continuous variable.

b p-value = Chi-Squared test for categorical variable.

### Serology results

Sampling took place between April 14, 2022, and November 16, 2022, with a mean interval of 221.91 ± 87.43 days (range: 77‒566 days) between the third dose and sampling. For infected patients, the mean interval between infection confirmation and sampling was 94.27 ± 65.25 days (range: 9‒261 days).

The median sVNT inhibition scores ( %) for BA.1, BA.2, and BA.4/5 were significantly higher in SARS-CoV-2-infected patients compared to uninfected patients. Specifically, infected patients had median scores of 58.11 % (Q1/Q3 = 9.26/76.18) for BA.1, 88.25 % (Q1/Q3 = 57.97/94.92) for BA.2, and 56.85 % (Q1/Q3 = 21.95/86.74) for BA.4/5. In contrast, uninfected patients had median scores of 5.89 % (Q1/Q3 = −2.78/30.74) for BA.1, 47.06 % (Q1/Q3 = 28.72/75.38) for BA.2, and 0.15 % (Q1/Q3 = −1.47/4.25) for BA.4/5 (Table 3). The presumed protection rates for each variant in uninfected patients were notably low: 12.9 % for BA.1, 32.3 % for BA.2, and 3.2 % for BA.4/5. To investigate potential differences in neutralizing antibody responses by vaccine type, sVNT inhibition scores were analyzed for homologous vector, homologous mRNA, and heterologous vaccine recipients in both infected and uninfected groups (Supplementary Table 2). No significant differences were observed among vaccine types (*p* > 0.05 for all subvariants), though the small sample size of the homologous vector vaccine group (*n* = 1 per group) limited statistical comparisons.

**Table 3:** Comparison of serology results between SARS-CoV-2-infected and uninfected subjects.

	SARS-CoV-2 uninfected subjects (*n* = 31)	SARS-CoV-2 infected subjects (*n* = 26)	p-value
**BA.1**			
**Seropositive, n (%)**	9 (29.03 %)	17(65.38 %)	0.0132^a^
*Presumed Protection Rates, n (%)	4(12.9 %)	10(38.46 %)	0.0544^a^
**sVNT inhibition (%)**			
Mean ± Std	15.62 ± 32.11	47.65 ± 39.01	0.0016^b^
Median	5.89	58.11	0.0025^c^
Q1, Q3	−2.78, 30.74	9.26, 76.18	
Range	−47.55 ∼ 91.15	−35.83 ∼ 98.38	
**BA.2**			
**Seropositive, n (%)**	22(70.97 %)	22(84.62 %)	0.3648^a^
*Presumed Protection Rates, n (%)	10 (32.26 %)	19 (73.08 %)	0.005^a^
**sVNT inhibition (%)**			
Mean ± Std	50.94 ± 29.60	68.77 ± 41.52	0.0734^b^
Median	47.06	88.25	0.0144^c^
Q1, Q3	28.72, 75.38	57.97, 94.92	
Range	5.93 ∼ 97.98	−43.80 ∼ 97.92	
**BA.4/5**			
**Seropositive, n (%)**	4(12.9 %)	19(73.08 %)	<0.001^a^
d**Presumed Protection Rates, n (%)**	1(3.23 %)	12(46.15 %)	<0.001^a^
**sVNT inhibition (%)**			
Mean ± Std	8.71 ± 21.93	52.71 ± 37.35	<0.001^b^
Median	0.15	56.85	<0.001^c^
Q1, Q3	−1.47, 4.25	21.95, 86.74	
Range	−6.41 ∼ 96.78	−11.10 ∼ 98.72	

sVNT, Surrogate Virus Neutralization Test; Std, Standard Deviation.

a p-value = Chi-Squared test for categorical variable.

b p-value = Two sample *t*-test.

c p-value = Wilcoxon rank sum test (Mann-Whitney *U* test) for continuous variable.

d An sVNT inhibition value ≥ 68 % suggests sufficient protection against COVID-19 infection.

**Fig. 2:**
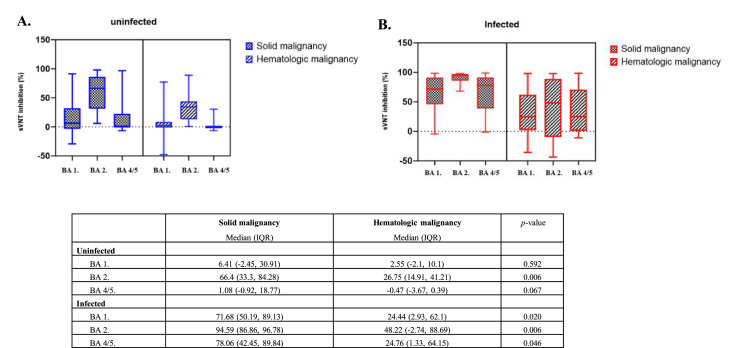
Neutralization effectiveness of BA.1, BA.2 and BA.4/5 in solid and hematological malignancies. Neutralizing antibody levels measured by percent inhibition of sVNT readings at (A) SARS-CoV-2 uninfected and (B) SARS-CoV-2 infected.

Among the infected patients, those with hematologic malignancies showed significantly lower median neutralizing antibody scores compared to patients with solid tumors for each Omicron subvariant: BA.1 (24.44 % hematologic vs. 71.68 % solid tumors, *p* = 0.020), BA.2 (48.22 % hematologic vs. 94.59 % solid tumors, *p* = 0.006), and BA.4/5 (24.76 % hematologic vs. 78.06 % solid tumors, *p* = 0.046) (Fig. 2). For uninfected patients, there were no significant differences in sVNT scores between solid and hematologic malignancies, except for BA.2. Neutralizing antibody responses were also evaluated by chemotherapy type to assess the impact of different regimens on vaccine efficacy (Supplementary Table 3). While no significant differences were observed across most chemotherapy types for BA.1 and BA.4/5 (*p* > 0.05), a trend toward higher sVNT scores was noted for BA.2 in patients receiving immune checkpoint inhibitors combined with chemotherapy (median 97.89 %), though the sample size was small (*n* = 3).

### Severity

During the study follow-up, three patients died: two due to cancer progression unrelated to COVID-19, and one following a treatment delay caused by SARS-CoV-2-related pneumonia, 91 days after the infection. Of the 26 patients with a history of SARS-CoV-2 infection, three developed pneumonia, and one experienced increased pleural effusion without pneumonia. Additionally, nine patients experienced delays in chemotherapy related to their infection, with an average delay of 17.75 ± 9.72 days.

## Discussion

The initial COVID-19 vaccination campaign in South Korea, which began on February 2, 2021, effectively reduced both disease incidence and mortality rates.^6^ However, the emergence of new variants, including the Omicron subvariants BA.4/5, coupled with the prolonged nature of the pandemic, led to a decrease in enthusiasm for receiving additional vaccine doses. In October 2022, South Korea introduced a bivalent mRNA COVID-19 vaccine, which included equal amounts of spike protein sequences from both the original wild-type strain and the BA.4/5 Omicron sublineage.^7^^,^^8^ Unfortunately, the campaign ended with a low uptake rate. This study aimed to evaluate the immune response of cancer patients to the Omicron variant and to assess the need for actively promoting additional vaccinations.^3^^,^^4^^,^^9^^,^^10^

In a previous study at the institution involving healthcare workers, the sVNT score for the Omicron variant (BA.1) remained high at 91.3 %, even 8-months after vaccination among those who had contracted the coronavirus post-third dose.^10^ However, in this study, cancer patients undergoing active chemotherapy who were infected after receiving the third vaccine dose showed a lower sVNT score of 58.1 %. This suggests that, despite adequate vaccination, cancer patients undergoing chemotherapy exhibit a weaker immune response compared to healthcare workers. The mean interval from vaccination to sample collection was 219.4 ± 86.1 days in both studies, indicating a similar time frame. The sVNT score in uninfected groups was low in both cases: 30.7 % for healthcare workers and 5.9 % for cancer patients. Although both groups had lower scores compared to the infected groups, cancer patients had significantly lower neutralizing antibody levels. This aligns with findings from a study during the Delta variant-dominant period, where the efficacy of the SARS-CoV-2 vaccine decreased more rapidly in cancer patients compared to healthy subjects after two doses.^11^^,^^12^ That study also noted a more rapid decline in sVNT values among patients with hematologic cancers. Similarly, in the present study, sVNT scores were significantly lower in the hematologic cancer group compared to the solid tumor group. Additionally, infection rates were 57.1 % in patients with hematologic cancers and 38.9 % in those with solid tumors, suggesting a potential correlation between rapid declines in immunogenicity and reduced vaccine efficacy. The weaker immune response observed in hematologic malignancies compared to solid tumors is likely attributable to two major factors:^13^ firstly, hematologic malignancies inherently compromise immune function by directly affecting immune cells such as B-cells and T-cells, which are critical for generating and maintaining robust antibody responses. Secondly, treatments for hematologic cancers frequently involve more intensive and aggressive chemotherapy or immunotherapy regimens (e.g., B-cell depletion therapies, high-dose corticosteroids, and intensive cytotoxic chemotherapy), significantly impairing immune response capacity. Collectively, these intrinsic and treatment-related factors likely contribute to rapid declines in immunogenicity, consequently reducing vaccine efficacy in hematologic cancer patients.

In this context, it is important to note that the presentation study intentionally adopted a real-world observational design, aiming to reflect clinical realities by including patients who naturally received diverse chemotherapy regimens tailored individually according to their cancer types and conditions. Thus, chemotherapy regimens and dosages were not strictly controlled, consistent with typical real-world clinical practice. Although the authors performed subgroup analyses according to chemotherapy regimen types (cytotoxic chemotherapy, targeted therapy, and immune checkpoint inhibitors; Supplementary Table 3), statistical power was limited due to small subgroup sizes, and no significant differences in neutralizing antibody responses were observed across these chemotherapy types. Similarly, subgroup analyses by vaccine type did not yield significant differences (Supplementary Table 2), though sample sizes for some groups were very small (e.g., homologous vector vaccine recipients, *n* = 2; immune checkpoint inhibitors combined with chemotherapy, *n* = 3), further limiting the ability to detect potential subgroup effects.

These findings emphasize the need for larger-scale studies to better explore how vaccine and chemotherapy regimens affect immunogenicity and to determine optimal vaccination intervals tailored to specific cancer types. Given these limitations and considering the significant vulnerability of patients with hematologic malignancies, actively recommending the administration of bivalent or newly developed vaccines remains crucial to reducing SARS-CoV-2 infection rates and preventing related complications in cancer patients undergoing chemotherapy.

A notable finding in this study was the unexpectedly high neutralizing antibody values for BA.2 (median 47.06 %) in patients without a recorded history of infection, compared to BA.1 (5.89 %) and BA.4/5 (0.15 %). This suggests possible under-detection of BA.2 infections, potentially due to milder systemic symptoms, such as lower fever incidence, as reported in Japan.^14^ Data from the Korea Disease Control and Prevention Agency (KDCA) also indicated fewer confirmed cases during the BA.2-dominant period compared to BA.1 and BA.5.^15^ The reduced frequency of systemic symptoms likely led to missed diagnoses, resulting in some patients developing antibodies without a recorded positive result. This highlights a limitation of symptom-based surveillance. Enhanced detection methods, such as routine wastewater testing or expanded asymptomatic testing, could mitigate such under-reporting and guide more timely booster updates.

This study is significant as it evaluated the neutralizing activity in the blood after the third SARS-CoV-2 vaccination in cancer patients undergoing chemotherapy and monitored the immunogenicity of newly emerged Omicron subvariants. However, it was conducted at a single center with a sample size of 57 patients, which limits the generalizability of the results. Additionally, the small sample sizes for certain subgroups, such as homologous vector vaccine recipients (*n* = 2) and patients receiving immune checkpoint inhibitors combined with chemotherapy (*n* = 3), restricted the statistical power of subgroup analyses, particularly for vaccine and chemotherapy type comparisons (Supplementary Tables 2 and 3). To further investigate vaccine efficacy, optimal vaccination intervals, and their relationship with cancer progression, future research should involve larger-scale cohort studies with more diverse cancer patient populations.

## Conclusion

In conclusion, this study reveals that cancer patients undergoing chemotherapy exhibit low neutralizing activity against Omicron subvariants BA.1, BA.2, and BA.4/5, even after receiving the third vaccination. This reduction is particularly notable among patients without a history of infection or those with hematologic cancers. Additionally, SARS-CoV-2 infections can cause delays in cancer treatment, potentially worsening disease progression. Therefore, it is crucial to actively recommend the administration of bivalent or newly developed vaccines to cancer patients in order to mitigate these risks.

## Funding

This research did not receive any specific grant from funding agencies in the public, commercial, or not-for-profit sectors.

## CRediT authorship contribution statement

**Jina Yun:** Conceptualization, Methodology, Investigation, Writing – original draft. **Bora Kim:** Investigation, Writing – original draft. **Hyuk Kim:** Formal analysis, Resources. **Sung Hee Lim:** Methodology, Formal analysis, Resources. **Seong Hyeok Choi:** Investigation. **Ji Youn Kim:** Investigation, Data curation. **Hyun Jung Kim:** Investigation, Data curation. **Seong Kyu Park:** Investigation, Supervision, Project administration.

## Declaration of competing interest

The authors declare no conflicts of interest.

## Appendix Supplementary materials

Image, application 1
