# Supplementary material for: Neutralizing activity against Omicron subvariants BA.1, BA.2, and BA.4/5 following the third SARS-CoV-2 vaccination in cancer patients undergoing chemotherapy
Source: Clinics (Sao Paulo). 2025 Aug 20;80:100757. doi: 10.1016/j.clinsp.2025.100757 (PMC12834060; doi:10.1016/j.clinsp.2025.100757)
Supplement: Supplementary file 1 [file mmc1.docx]

CLINICS-D-24-01187_Supplementary Material

**Supplementary Table 1** Clinical characteristics by vaccine type (homologous vector, homologous mRNA, heterologous).

| **Characteristic** | **Homologous vector vaccine**  **(n = 2)** | **Homologous mRNA vaccine**  **(n = 27)** | **Heterologous vaccine**  **(n = 28)** | **p-value** |
| --- | --- | --- | --- | --- |
| **Age, years** (Mean ± SD) | 63.00 ± 1.41 | 57.11 ± 13.26 | 66.68 ± 5.83 | 0.0166^a^ |
| **Sex (Male %)** | 1 (50%) | 14 (51.85%) | 19 (67.86%) | 0.4623^c^ |
| **SARS-Cov-2 Vaccine** |  |  |  | 0.4651^c^ |
| Uninfected | 1 (50.00%) | 17 (62.96%) | 13 (46.43%) |  |
| Infected | 1 (50.00%) | 10 (37.04%) | 15 (53.57%) |  |
| **Solid malignancies** | 0 (0.00%) | 17 (62.96%) | 19 (67.86%) | 0.1577^c^ |
| **Hematologic malignancies** | 2 (100.00%) | 10 (37.04%) | 9 (32.14%) |  |
| **Vaccination-sample interval (Q1, Q3)** | 258.0 (217.50, 298.50) | 223.0 (168.50, 255.00) | 200.0 (157.50, 245.00) | 0.6665^b^ |
| **Chemotherapeutic agents** |  |  |  | 0.9737^c^ |
| Cytotoxic chemotherapy | 0 (0.00%) | 8 (29.63%) | 9 (32.14%) |  |
| Targeted therapy | 1 (50.00%) | 7 (25.93%) | 5 (17.86%) |  |
| Immune checkpoint inhibitor (ICI) | 0 (0.00%) | 1 (3.70%) | 2 (7.14%) |  |
| Targeted therapy + chemotherapy | 1 (50.00%) | 8 (29.63%) | 9 (32.14%) |  |
| ICI + chemotherapy | 0 (0.00%) | 2 (7.41%) | 1 (3.57%) |  |
| Others | 0 (0.00%) | 1 (3.70%) | 2 (7.14%) |  |

^a^ p-value = One-way ANOVA for continuous variable.

^b^ p-value = Kruskal-Wallis test for continuous variable.

^c^ p-value = Chi-Squared test for categorical variable.

**Supplementary Table 2** sVNT inhibition scores by vaccine type for both infected and uninfected groups.

|  | **SARS-CoV-2 uninfected subjects (n = 31)** | | | **p-value** | **SARS-CoV-2 infected subjects (n = 26)** | | | **p-value** |
| --- | --- | --- | --- | --- | --- | --- | --- | --- |
|  | **Homologous vector vaccine**  **(n = 1)** | **Homologous mRNA vaccine**  **(n = 17)** | **Heterologous vaccine**  **(n = 13)** |  | **Homologous vector vaccine**  **(n = 1)** | **Homologous mRNA vaccine**  **(n = 10)** | **Heterologous vaccine**  **(n = 15)** |  |
| **BA.1** |  |  |  |  |  |  |  |  |
| **sVNT inhibition (%)** |  |  |  |  |  |  |  |  |
| Mean ± Std | 10.10 ± N/A | 20.76 ± 31.07 | 9.33 ± 34.75 | NA | -10.71 ± N/A | 55.93 ± 31.07 | 46.01 ± 42.44 | NA |
| Median | 10.10 | 6.93 | 2.55 | 0.6458 | -10.71 | 62.13 | 48.87 | 0.2912 |
| Q1, Q3 | 10.10, 10.10 | -0.74, 48.51 | -11.33, 30.40 |  | -10.71, -10.71 | 49.30, 68.72 | 11.86, 80.33 |  |
| Range | 10.10~ 10.10 | -12.39 ~ 79.31 | -47.55 ~ 91.15 |  | -10.71~ -10.71 | 3.39 ~ 97.23 | -35.83~ 98.38 |  |
| **BA.2** |  |  |  |  |  |  |  |  |
| **sVNT inhibition (%)** |  |  |  |  |  |  |  |  |
| Mean ± Std | 46.32 ± N/A | 49.29 ± 31.31 | 53.45 ± 29.51 | NA | -43.80 ± N/A | 77.14 ± 30.27 | 70.70 ± 39.12 | NA |
| Median | 46.32 | 41.21 | 50.86 | 0.9067 | -43.80 | 87.83 | 88.85 | 0.2462 |
| Q1, Q3 | 46.32, 46.32 | 29.56, 82.02 | 27.87, 68.74 |  | -43.80, -43.80 | 72.73, 95.91 | 59.45, 94.58 |  |
| Range | 46.32~ 46.32 | 5.93 ~ 97.98 | 13.66 ~ 97.89 |  | -43.80~ -43.80 | 0.69 ~ 97.61 | -15.51~ 97.92 |  |
| **BA.4/5** |  |  |  |  |  |  |  |  |
| **sVNT inhibition (%)** |  |  |  |  |  |  |  |  |
| Mean ± Std | 1.38 ± N/A | 7.17 ± 18.16 | 11.29 ± 27.34 | NA | -11.10 ± N/A | 57.09 ± 35.44 | 54.05 ± 37.11 | NA |
| Median | 1.38 | -0.47 | 2.55 | 0.0343 | -11.10 | 65.53 | 52.83 | 0.2494 |
| Q1, Q3 | 1.38, 1.38 | -1.86, 1.64 | 0.02, 4.57 |  | -11.10, -11.10 | 41.15, 83.74 | 24.77, 88.35 |  |
| Range | 1.38 ~ 1.38 | -6.41 ~ 50.57 | -6.41 ~ 96.78 |  | -11.10~ -11.10 | -1.27 ~ 96.88 | -1.83 ~ 98.72 |  |

sVNT, Surrogate Virus Neutralization Test; Std, Standard deviation.

p-value = Kruskal-Wallis test for continuous variable.

Note: Comparisons involving the homologous vector vaccine group (n = 1) should be interpreted with caution due to the small sample size, limiting statistical power.

**Supplementary Table 3** sVNT scores by chemotherapy type.

|  | **Cytotoxic chemotherapy**  **(n = 17)** | **Targeted therapy**  **(n = 13)** | **Immune checkpoint inhibitor (ICI)**  **(n = 3)** | **Targeted therapy + chemotherapy**  **(n = 18)** | **ICI + chemotherapy**  **(n = 3)** | **Others**  **(n = 3)** | **p-value** |
| --- | --- | --- | --- | --- | --- | --- | --- |
| **BA.1** |  |  |  |  |  |  |  |
| **sVNT inhibition (%)** |  |  |  |  |  |  |  |
| Mean ± Std | 31.40 ± 41.33 | 43.32 ± 36.21 | 43.90 ± 42.37 | 11.29 ± 26.83 | 73.57 ± 21.05 | 23.52 ± 65.78 | 0.0541^a^ |
| Median | 30.40 | 54.15 | 48.87 | 5.88 | 79.31 | 2.55 | 0.1231^b^ |
| Q1, Q3 | 0.57, 64.92 | 7.02, 69.52 | 24.07, 66.22 | -1.19, 22.84 | 64.78, 85.23 | -13.33, 49.89 |  |
| Range | -47.55 ~ 98.38 | -10.71 ~ 97.97 | -0.74 ~ 83.56 | -35.83 ~ 91.82 | 50.24 ~ 91.15 | -29.21 ~ 97.23 |  |
| **BA.2** |  |  |  |  |  |  |  |
| **sVNT inhibition (%)** |  |  |  |  |  |  |  |
| Mean ± Std | 70.57 ± 27.50 | 46.30 ± 48.39 | 70.89 ± 27.14 | 50.99 ± 33.71 | 92.63 ± 9.19 | 52.38 ± 40.22 | 0.0311^a^ |
| Median | 86.52 | 54.31 | 77.02 | 56.33 | 97.89 | 38.88 | 0.1791^b^ |
| Q1, Q3 | 49.31, 90.42 | 26.75, 94.12 | 59.12, 85.73 | 17.21, 78.94 | 89.95, 97.94 | 29.77, 68.25 |  |
| Range | 14.91 ~ 97.92 | -43.80 ~ 97.84 | 41.21 ~ 94.45 | 0.69 ~ 96.55 | 82.02 ~ 97.98 | 20.65 ~ 97.61 |  |
| **BA.4/5** |  |  |  |  |  |  |  |
| **sVNT inhibition (%)** |  |  |  |  |  |  |  |
| Mean ± Std | 39.71 ± 37.87 | 25.88 ± 36.91 | 36.86 ± 37.83 | 15.66 ± 31.73 | 46.36 ± 48.89 | 32.47 ± 55.78 | 0.6194^a^ |
| Median | 37.71 | 1.62 | 35.53 | 1.51 | 43.15 | 0.39 | 0.5113^b^ |
| Q1, Q3 | 2.55, 73.94 | -1.83, 50.57 | 17.62, 55.44 | -1.19, 16.82 | 21.15, 69.97 | 0.27, 48.63 |  |
| Range | -1.88 ~ 98.72 | -11.10 ~ 98.31 | -0.29 ~ 75.34 | -6.41 ~ 94.30 | -0.85 ~ 96.78 | 0.15 ~ 96.88 |  |

^a^ p-value = One-way ANOVA.

^b^ p-value = Kruskal-Wallis test for continuous variable.
